# Metabolic tumor burden on postsurgical PET/CT predicts survival of patients with gastric cancer

**DOI:** 10.1186/s40644-019-0205-9

**Published:** 2019-03-22

**Authors:** Gaofeng Sun, Chao Cheng, Xiao Li, Tao Wang, Jian Yang, Danni Li

**Affiliations:** Department of Nuclear Medicine, Changhai Hospital, Block 10, Changhai hospital, No. 168 in Changhai Road, Yangpu district, Shanghai, 200433 China

**Keywords:** Gastric cancer, Prognosis, 2-[18F] Fluoro-2-deoxy-D-glucose (^18^F-FDG), Positron emission tomography/computed tomography (PET/CT), Metabolic tumor burden

## Abstract

**Background:**

The prognostic value of postoperative ^18^F-fluorodeoxyglucose (^18^F-FDG) positron emission tomography/computed tomography (PET/CT) to patients with gastric cancer remains unclear. This study aims to investigate the prognostic value of whole body (WB) metabolic tumor burden (MTB_WB_) on postsurgical ^18^F-FDG PET/CT to patients with gastric cancer.

**Methods:**

A total of 376 patients with surgeries-confirmed gastric cancer were enrolled. Clinicopathologic information, overall survival (OS) and MTB_WB_ parameters on postsurgical PET/CT, in terms of WB maximum standardized uptake value (SUV_WBmax_), WB metabolic tumor volume (MTV_WB_), and WB total lesion glycolysis (TLG_WB_) were collected. In-between differences of patient clinicopathologic characteristics, OS and MTB_WB_ measurements were compared using chi-square test, Fisher’s exact test, Student’s t test or the Kaplan-Meier survival analysis. The optimal cutoffs of MTB_WB_ measurements were calculated through the receiver operating characteristic (ROC) curve analysis. Univariable and multivariable Cox proportional hazard regression were performed to test the predictive value of the clinicopathologic factors and MTB_WB_ measurements to patient survival.

**Results:**

The PET-positive patients had significantly decreased OS based on either Kaplan-Meier survival analysis (*P* <  0.001) or univariable Cox regression (hazard ratio [HR] = 2.850, *P* <  0.001). In patients with PET-positive tumors, the associations between OS and SUV_WBmax_, MTV_WB_ and TLG_WB_ were significant, both in univariable analysis (*P* <  0.001, *P* <  0.001 and *P* = 0.001, respectively) and in multivariable analysis (*P* = 0.002, *P* <  0.001 and *P* = 0.005, respectively). Patient OS among groups dichotomized by cutoffs of SUV_WBmax_ > 8.6, MTV_WB_ > 91.5 cm^3^, and TLG_WB_ > 477.6 cm^3^ were significantly different (*P* = 0.001, *P* <  0.001 and *P* = 0.001, respectively).

**Conclusions:**

MTB_WB_, in terms of SUV_WBmax_, MTV_WB_ and TLG_WB_, on postsurgical ^18^F-FDG PET/CT provides prognostic value to patients with gastric cancer after curative resection.

## Background

Gastric cancer is the fifth most common cancer worldwide [1]. There are about 7.4 new cases of gastric cancer per 100,000 men and women per year in United States [2]. The prognosis of gastric cancer is dismal, with the 5-year survival rate being about 20–25%, ranking the second leading cause of cancer-related death [3]. The Asian population exhibit the highest mortality rate which was estimated 24 per 100,000 men and 9.8 per 100,000 women [4]. Surgical resection of cancer lesions with lymph node dissection is the only curative treatment, but survival rate is till unsatisfactory even when achieving R0 resection. About 28–47% patients experienced recurrences after operation, which had commonly led to death [5–8]. Therefore, a practical method that can precisely predict the survival outcomes of patients with gastric cancer is essential, because stratification of patients with potential survival outcomes may influence the treatment decision.

In recent years, 2-[18F] Fluoro-2-deoxy-D-glucose (^18^F-FDG) positron emission tomography/computed tomography (PET/CT) has demonstrated significant capacity in staging, detecting recurrence, evaluating treatment response, and predicting prognosis of gastric-cancer patients [5, 6, 9–13]. A unique advantage of ^18^F-FDG PET/CT is the ability to provide the quantitative information of FDG uptake for assessing tumor glucose metabolism. Among the various quantitative parameters, the maximum standardized uptake value (SUV_max_) from a single pixel anywhere within the tumor is the most commonly used. However, it cannot accurately reflect the overall metabolic activity of tumor. In contrast, the volume-based parameters such as the metabolic tumor volume (MTV) and the total lesion glycolysis (TLG) evaluate global volume and metabolism. MTV is defined as the sum of metabolic volume above a certain threshold, while TLG is defined as the product of tumor volume and mean metabolic activity within the tumor [11, 14]. MTV and TLG have demonstrated excellent performance in predicting clinical outcomes of patients with gastric cancer in previous studies [9, 14, 15].

Clinically, patients with a single metastasis are treated equally with those having multiple metastases, which may be partially due to the difficulty in quantifying metastatic burden. In this regard, MTV and TLG have additional advantage of summing multiple lesions together, and therefore are suitable in quantifying whole body metabolic tumor burden (MTB_WB_) of cancer patient. Actually, previous studies have demonstrated better correlation of MTB_WB_ with survival of cancer patients, including gastric cancer, as compared to SUV [14, 16]. Besides, MTB_WB_ is relatively immune to the effect of inter-observer variability.

Park et al. had demonstrated that MTB_WB_ facilitated stratification of stage IV recurrent or metastatic gastric cancer patients and allowed to improve prediction of chemotherapy response and prognosis even after considering HER2 status, but this study was based on preoperative ^18^F-FDG PET/CT [14]. Although MTB_WB_ on postoperative ^18^F-FDG PET/CT had demonstrated its prognostic value in patients with lung cancer by Zhang et al. [16], its potential utility for gastric cancer patients remains unclear. As far as we know, there are a substantial portion of surgical patients who only underwent postsurgical PET/CT examination for recurrence surveillance. In this study, we attempted to prove our hypothesis that MTB_WB_ on postsurgical ^18^F-FDG PET/CT correlated with survival of patients with gastric cancer.

## Materials and methods

### Patients enrollment

This study was approved by the institutional ethics committee in accordance with the Helsinki Declaration of 1975, revised in 2000. Informed consents were obtained from all patients.

We firstly reviewed the electronic registry system and medical records in the PET/CT center of our hospital. A total of 1554 patients with gastric cancer who underwent surgical resection between 2011 and 2012 were searched out. Thereafter, 1178 cases were excluded due to one of the following conditions: 1) PET/CT were performed before surgeries (*n* = 649); 2) surgeries were not curative intent (*n* = 258); 3) PET/CT were performed after chemo- or radio-therapy (*n* = 220); 4) they had a second primary malignant tumor (*n* = 51). Finally, 376 gastric-cancer subjects with post-surgery PET/CT were included in this study. All PET/CT examinations were performed for evaluation of suspected recurrence.

### PET/CT acquisition

All patients were fasted for at least 6 h and serum glucose levels were checked before injection of ^18^F-FDG (3.7 MBq/kg). The amount of injected radioactivity was routinely calculated by measuring the radioactivity of the syringe before and after injection. The mean injected ^18^F-FDG dose was 307.1 MBq (range 204.5–488.4 MBq). PET/CT scanning was performed about 1 h after intravenous injection of ^18^F-FDG. In cases with no symptoms of gastric obstruction, patients were instructed to drink at least 500 mL of water. Then, PET/CT scanning was performed using a hybrid Biograph 64 Truepoint PET/CT scanner (Siemens Healthcare, Knoxville, TN, United States) from the skull base to the proximal thigh in a supine position. Helical CT acquisitions were performed firstly without contrast enhancement using the following parameters: tube current, 200 mAs; tube voltage, 120 kV; collimation configuration, 64 × 0.6 mm; matrix size, 512 × 512; scanning time, 0.8 s per rotation. For review, the CT images were reconstructed with a slice thickness of 1.5 mm and an increment of 1.25. PET acquisition time was 2 min/bed position in three-dimensional mode, and images were reconstructed by using time-of-flight ordered-subsets expectation maximization iterative method. The image matrix was 256 × 256, corresponding to a 3-mm in-plane pixel size with a plane thickness of 3 mm. If patient had multiple postsurgical PET/CT, the first one was used for analysis. The median time from curative surgery to the postsurgical PET/CT scan was 8 months (range, 2–18).

### Imaging analysis

Two board-certified observers (A: **G.S.**, B: **C.C.**) who had more than 5 years in reading PET/CT images performed the evaluation of PET/CT images independently in two steps. Firstly, PET/CT images were visually assessed to classify patients into positive or negative with respect to malignancy related ^18^F-FDG uptake. Lesions showing focally increased ^18^F-FDG uptake exceeding the uptake of surrounding normal tissue and corresponding to cancer lesions on contrast-enhanced CT images or gastroscopies were read as positive ^18^F-FDG uptake, otherwise judged to be negative ^18^F-FDG uptake. Secondly, for patients with ^18^F-FDG positive lesions, measurements of MTB_WB_ on PET/CT were performed according to the method described in prior studies [16], using the commercially available PET Edge tool (MIMvista, Cleveland, Ohio). The volume of interest was created using an isocontour threshold of SUV ≥ 2.5 for lesions with SUV_max_ > 2.5, but for lesions with a SUV_max_ ≤ 2.5, a 40% SUV_max_ threshold was used [14]. Each VOI generated a SUV_max_, TLG, and MTV. Whole body SUV_max_ (SUV_WBmax_) was defined as the single highest SUV_max_ among all lesions, while MTV_WB_ and TLG_WB_ were the sum of MTV and TLG of all lesions, respectively. For testing reproducibility, observer A performed the MTB_WB_ measurements twice with an interval of more than two months.

### Clinical and pathological information

Clinical data including patient gender, age, pathological findings, surgical procedures and treatment after the PET/CT examination were collected from the medical records. Histopathologically, the primary tumors were classified into 2 microscopic growth types on the basis of the Lauren classification: intestinal and non-intestinal. Diffuse, mixed, and unclassifiable types were included in non-intestinal type. In addition, the primary tumors were also categorized into papillary adenocarcinoma (PAC), well differentiated and moderately differentiated tubular adenocarcinoma (TAC), poorly differentiated adenocarcinoma (PDAC), signet-ring cell carcinoma (SRC) and mucinous adenocarcinoma (MAC) according to the Japanese classification of gastric cancer [17].

### Follow up

All patients underwent routine clinical follow up including history taking, physical examination, serological tumor marker testing, contrast-enhanced abdominopelvic CT or MRI scanning, and gastroduodenoscopy. In the first 3 years after operation, all patients were clinically assessed every 3–4 months. Afterwards, the patients were assessed every 4–6 months. Clinical restaging was performed to patient based on the follow-up diagnosis, including locoregional recurrence, distant metastasis and none. The mean duration of follow up was 31.2 ± 13.4 (range 8–60) months. If the clinical assessment or diagnostic study showed an abnormal finding, additional pathological confirmation was performed. The overall survival (OS) was the primary endpoint which was defined as the time interval from the first postsurgical PET/CT scan to the date the patients died of any cause. Patients last known to be alive were censored at the date of last contact.

### Statistical analysis

Patients were classified as ^18^F-FDG PET/CT positive and negative. Inter-observer variabilities of MTB_WB_ measurements between observer A and B, and intra-observer variabilities between two-time measurements performed by observer A were evaluated by calculating intraclass correlation coefficients (ICC) for the PET-positive patients. An ICC greater than 0.75 indicates good agreement [18]. Clinicopathologic characteristics of patients and the MTB_WB_ measurements were compared between positive and negative patients using chi-square test, Fisher’s exact test and Student’s t test. Kaplan-Meier survival analysis with the log-rank test was performed to compare cumulative survival rates between patients with positive or negative ^18^F-FDG uptake. For investigating association between MTB_WB_ and OS, patients with positive ^18^F-FDG uptake were divided by the optimal cutoffs of MTB_WB_ measurement through the receiver operating characteristic (ROC) curve analysis in predicting patient death. The criterion for determining optimal cutoff point was the maximum Youden index. The predictive values of the clinicopathologic factors and MTB_WB_ measurements were analyzed with the univariable and multivariable Cox proportional hazard regression. Those variables with *P* <  0.05 were included for multivariable analysis. A backward stepwise selection model was used in the multivariable analysis to address the problem of multicolinearity. The Harrell C concordance statistic (range, 0–1) was calculated to evaluate the power of the Cox model. All statistical analyses were performed using SPSS 20 (IBM SPSS Inc., Chicago, IL, USA) and all hypothesis tests were two sided with a significance level of 0.05.

## Results

### Clinicopathologic characteristics of patients

Of the 376 patients included, 180 (47.9%) were men and 196 (52.1%) were women, with a median age of 50 (range, 21–83) years. The mean interval between injection of ^18^F-FDG and PET/CT scanning was 63.2 ± 15.6 min. On postsurgical ^18^F-FDG PET/CT, 236 (62.8%) patients were FDG-uptake positive while 140 (37.2%) patients were negative. Comparisons of clinicopathologic characteristics between FDG-uptake positive and negative patients were summarized in Table 1. Pathologically, patients that were FDG-uptake positive had higher rates of PAC/TAC, Lauren intestinal subtypes and lymphovascular invasion but a lower rate of SRC/MAC of primary tumor than negative patients (*P* <  0.05). The T stages of primary tumor were also relatively higher in patients with positive FDG uptake compared to negative patients (*P* <  0.001). Two hundred and thirty-six patients experienced tumor recurrences or metastases, all of whom had FDG-positive tumors. Consequently, more patients in FDG-uptake positive group underwent postsurgical adjuvant treatments than negative patients (*P* <  0.001). Specifically, in FDG-uptake positive group, 139 patients underwent chemotherapy and 78 patients underwent radiotherapy including 42 patients underwent both therapy; while in FDG-negative group, 33 patients experienced chemotherapy and 19 patients experienced radiotherapy including 7 patients experienced both therapy. The treatment types were not significantly different between FDG-uptake positive group and negative group (*P* = 0.430), neither was age, gender ratio, gastrectomy process and time intervals from surgeries to PET/CT scanning.

**Table 1:** Characteristics of patients between PET positive and negative groups

Characteristics	All patients (*n* = 376)	PET findings		*P* values
		Positive (*n* = 236)	Negative (*n* = 140)	
Age (mean ± SD)	51.1 ± 18.4	51.4 ± 18.1	50.7 ± 18.9	0.734^*^
Gender				0.827^†^
Men (%)	180 (47.9%)	114 (48.3%)	66 (47.1%)	
Women (%)	196 (52.1%)	122 (51.7%)	74 (52.9%)	
Pathological type of primary tumor				< 0.001^†^
PAC/TAC	112 (29.8%)	83 (35.2%)	29 (20.7%)	
PDAC	180 (47.9%)	117 (49.6%)	63 (45.0%)	
SRC/MAC	84 (22.3%)	36 (15.3%)	48 (34.3%)	
Lauren classifications				0.020^†^
Intestinal	130 (34.6%)	92 (39.0%)	38 (27.1%)	
Non-intestinal	246 (65.4%)	144 (61.0%)	102 (72.9%)	
T stage of primary tumor				< 0.001^†^
T1	95 (25.3%)	37 (15.7%)	58 (41.4%)	
T2	112 (29.8%)	75 (31.8%)	37 (26.4%)	
T3	133 (35.4%)	101 (42.8%)	32 (22.9%)	
T4	36 (9.6%)	23 (9.7%)	13 (9.3%)	
Gastrectomy				0.484^†^
Total	178 (47.3%)	115 (48.7%)	63 (45.0%)	
Subtotal	198 (52.7%)	121 (51.3%)	77 (55.0%)	
Lymphovascular invasion				< 0.001^†^
Yes	195 (51.9%)	146 (61.9%)	49 (35.0%)	
No	181 (48.1%)	90 (38.1%)	91 (65.0%)	
Clinical restage				< 0.001^†^
Locoregional recurrence	140 (37.2%)	140 (59.3%)	…	
Distant metastasis	96 (25.6%)	96 (40.7%)	…	
None	140 (37.2%)	…	140 (100%)	
Postsurgical adjuvant treatment				< 0.001^†^
Yes	220 (58.5%)	175 (74.2%)	45 (32.1%)	
No	156 (41.5%)	61 (25.8%)	95 (67.9%)	
Intervals between surgery and PET/CT (month)	8.6 ± 4.2	8.3 ± 4.2	9.1 ± 4.1	0.103^*^
Postsurgical MTB_WB_ on PET/CT				…
SUV_WBmax_	…	8.9 ± 4.7	…	
MTV_WB_ (cm^3^)	…	289.7 ± 256.3	…	
TLG_WB_ (cm^3^)	…	1002.1 ± 733.6	…	
Median OS (month)	19 (5–60)	16 (5–59)	29 (12–60)	< 0.001^‡^

Note. *SD* standard deviation, *PAC* papillary adenocarcinoma, *TAC* tubular adenocarcinoma, *PDAC* poorly differentiated adenocarcinoma, *SRC* signet-ring cell carcinoma, *MAC* mucinous adenocarcinoma, *MTB* metabolic tumor burden, *WB* whole body, *PET/CT* positron emission tomography/computed tomography, *SUV* standardized uptake value, *Max* maximum, *MTV* metabolic tumor volume, *TLG* total lesion glycolysis, *OS* overall survival. Data in parentheses are percentages or ranges of corresponding variables. *, Student’s t test; †, Chi square test; ‡, Kaplan-Meier analysis with the log-rank test

The intra- and inter-observer agreements of MTB_WB_ measurements expressed as ICCs were good, with the former ranging from 0.857 for TLG_WB_ to 0.912 for SUV_WBmax_, and the latter ranging from 0.861 for TLG_WB_ to 0.905 for SUV_WBmax_ (Table 2).

**Table 2:** Intra- and Inter-observer agreements of the MTB_WB_ measurements on PET/CT expressed as intraclass correlation coefficients

Agreements	SUV_WBmax_	MTV_WB_	TLG_WB_
Intraobserver	0.912 (0.863–0.936)	0.875 (0.812–0.912)	0.857 (0.807–0.891)
Interobserver	0.905 (0.887–0.931)	0.882 (0.854–0.903)	0.861 (0.811–0.902)

Note: Data in parentheses are 95% confidential intervals. *MTB* metabolic tumor burden, *WB* whole body, *PET/CT* positron emission tomography/computed tomography, *SUV* standardized uptake value, *Max* maximum, *MTV* metabolic tumor volume, *TLG* total lesion glycolysis

### Correlation between presence of FDG-uptake tumors and OS

**Fig. 1:**
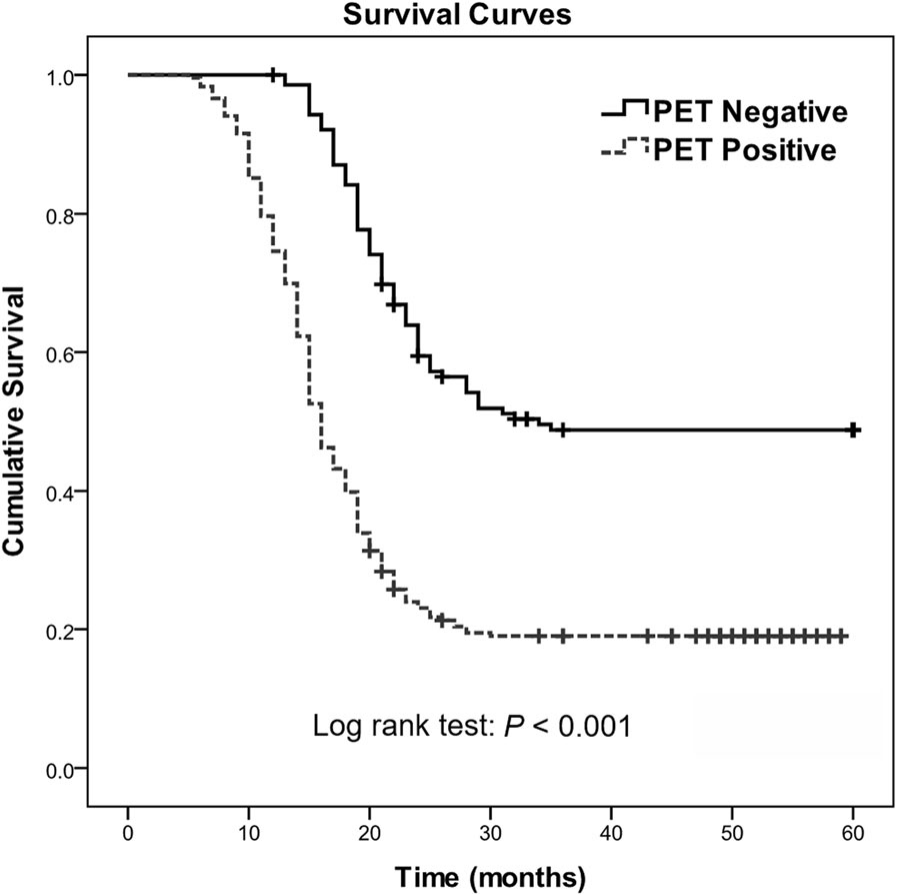
Kaplan-Meier curves of OS in the whole patient cohort (*n* = 376) grouped according to FDG-uptake positive and negative

Significantly worse survival was found in patients with positive FDG uptake, compared to patients with negative FDG uptake (*P* <  0.001; Fig. 1). Univariable Cox regression analysis showed a notably shorter OS in patients with positive FDG uptake (hazard ratio [HR] = 2.850, *P* <  0.001) than patients with negative FDG uptake (Table 3). The median OS in patients with positive FDG uptake was much shorter than those of patients with negative FDG uptake (16 vs. 29 months, *P* <  0.001; Table 1).

**Table 3:** Univariable Cox regression analysis for associations between clinicopathologic characteristics and OS in the whole study cohort (*n* = 376)

Variables	Number	HR	95% CI	*P* values
Age (years)				
≥ 50	192 (51.1%)	1.019	0.799–1.300	0.877
< 50^a^	184 (48.9%)	1.000		
Gender				
Men	180 (47.9%)	0.993	0.778–1.267	0.955
Women^a^	196 (52.1%)	1.000	…	…
Pathological type of primary tumor				
PAC/TAC	112 (29.8%)	1.122	0.797–1.579	0.509
PDAC	180 (47.9%)	1.115	0.815–1.526	0.495
SRC/MAC^a^	84 (22.3%)	1.000	…	…
Lauren classifications				
Intestinal	130 (34.6%)	1.125	0.873–1.451	0.363
Non-intestinal^a^	246 (65.4%)	1.000	…	…
T stage of primary tumor				
T1	95 (25.3%)	1.005	0.611–1.654	0.984
T2	112 (29.8%)	1.508	0.933–2.437	0.094
T3	133 (35.4%)	1.515	0.946–2.428	0.084
T4^a^	36 (9.6%)	1.000	…	…
Gastrectomy				
Total	178 (47.3%)	0.899	0.705–1.147	0.392
Subtotal^a^	198 (52.7%)	1.000	…	…
Lymphovascular invasion				
Yes	195 (51.9%)	1.482	1.159–1.895	0.002
No^a^	181 (48.1%)	1.000	…	…
Clinical restage				
Locoregional recurrence	140 (37.2%)	0.968	0.725–1.291	0.823
Distant metastasis	96 (25.6%)	0.344	0.249–0.476	< 0.001
None^a^	140 (37.2%)	1.000	…	…
Postsurgical adjuvant treatment				
Yes	220 (58.5%)	1.552	1.207–1.997	0.001
No^a^	156 (41.5%)	1.000	…	…
PET finding				
Positive	236 (62.8%)	2.850	2.161–3.758	< 0.001
Negative^a^	140 (37.2%)	1.000	…	…

Note. *HR* hazard ratio, *CI* confidence interval, *PAC* papillary adenocarcinoma, *TAC* tubular adenocarcinoma, *PDAC* poorly differentiated adenocarcinoma, *SRC* signet-ring cell carcinoma, *MRC* mucinous adenocarcinoma, *PET* positron emission tomography. ^a^, Reference group. Data in parentheses are percentages of corresponding variables

Other characteristics that were significantly associated with OS included clinical restage (*P* <  0.001), lymphovascular invasion (HR = 1.482, *P* = 0.002), and postsurgical adjuvant treatment (HR = 1.552, *P* = 0.001). The median OS of patients with primary tumor of T1-T4 stage were 23, 18, 18 and 21 months respectively; whereas the median OS of patients with none recurrence, locoregional recurrence and distant metastases were 29, 16, and 16 months, respectively. Patient age, gender, pathological subtype, Lauren classification, and type of surgery were not related to OS (Table 3).

### Correlations between quantitative MTB_WB_ and OS

**Fig. 2:**
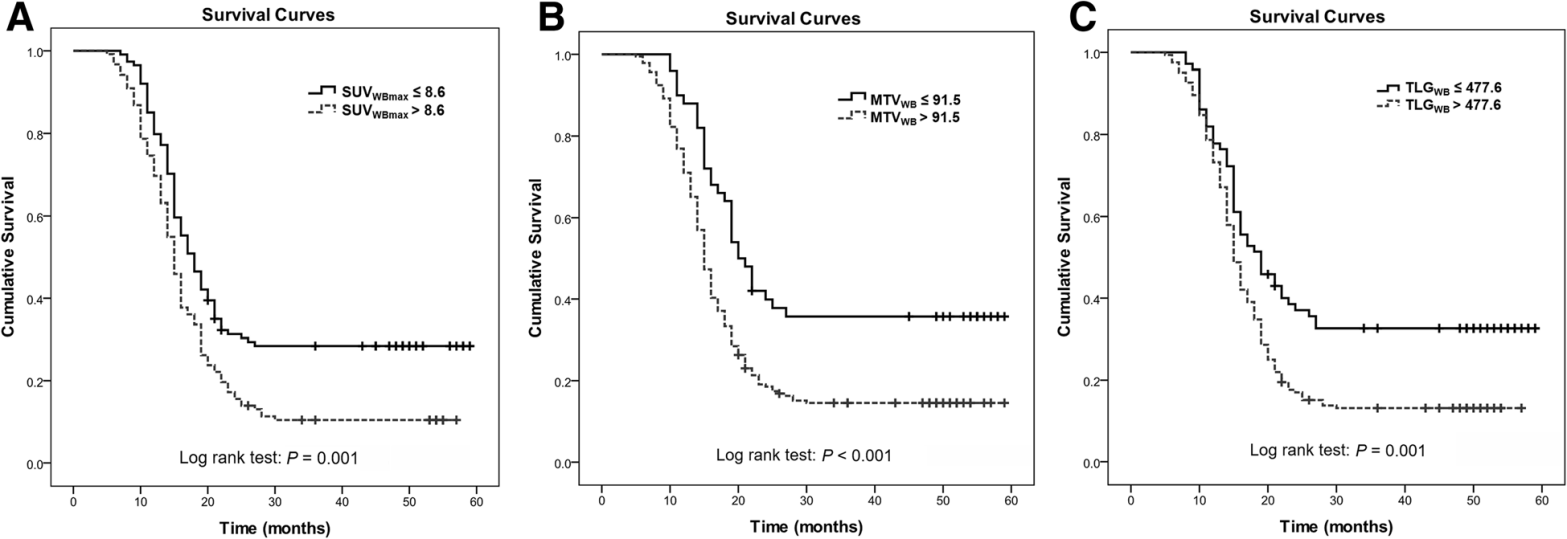
Kaplan-Meier curves of OS in patients with positive FDG uptake (*n* = 236). Patients were grouped by cutoffs of SUV_WBmax_ > 8.6 (**a**), MTV_WB_ > 91.5 cm^3^ (**b**), and TLG_WB_ > 477.6 cm^3^ (**c**)

Within FDG-positive patient group, only quantitative MTB_WB_ measurements were identified notably correlated with OS, based on univariable analysis (Table 4). The HRs of SUV_WBmax_, MTV_WB_ and TLG_WB_ were 1.066 (*P* <  0.001), 1.421 (*P* <  0.001) and 1.301 (*P* = 0.001), respectively. In multivariable analysis, these three parameters were all independent variables that significantly predicted worse OS in patients with gastric cancer (Table 4). The HRs were 1.052 (*P* = 0.002) for SUV_WBmax_, 1.443 (*P* <  0.001) for MTV_WB_, and 1.332 (*P* = 0.005) for TLG_WB_. The Harrell C concordance statistic was 0.828, indicating a high goodness of fit of the regression model. Based on ROC curve analyses, the optimal cutoffs of SUV_WBmax_, MTV_WB_ and TLG_WB_ for predicting patient death were > 8.6, > 91.5 cm^3^ and > 477.6 cm^3^, respectively. The differences of OS between groups dichotomized by these cutoffs were significant (*P* = 0.001 for SUV_WBmax_, *P* <  0.001 for MTV_WB_, and *P* = 0.001 for TLG_WB_), demonstrated by the Kaplan-Meier analysis with Log rank test (Fig. 2).

**Table 4:** Univariable and multivariable Cox regression analysis for associations between clinicopathologic characteristics and OS in patients with positive PET findings (*n* = 236)

Variables	Number	Univariable		Multivariable	
		HR	*P* values	HR	*P* values
Age (years)					
≥ 50	123 (52.1%)	0.971	0.838		
< 50^a^	113 (47.9%)	1.000			
Gender					
Men	114 (48.3%)	0.973	0.851		
Women^a^	122 (51.7%)	1.000			
Pathological type of primary tumor					
PAC/TAC	83 (35.2%)	0.832	0.405		
PDAC	117 (49.6%)	0.964	0.860		
SRC/MAC^a^	36 (15.3%)	1.000			
Lauren classifications					
Intestinal	92 (39.0%)	1.047	0.757		
Non-intestinal^a^	144 (61.0%)	1.000			
T stage of primary tumor					
T1	37 (15.7%)	1.046	0.884		
T2	75 (31.8%)	1.381	0.239		
T3	101 (42.8%)	1.235	0.428		
T4^a^	23 (9.7%)	1.000			
Gastrectomy					
Total	115 (48.7%)	0.982	0.899		
Subtotal^a^	121 (51.3%)	1.000			
Lymphovascular invasion					
Yes	146 (61.9%)	1.334	0.060		
No^a^	90 (38.1%)	1.000			
Clinical restage					
Locoregional recurrence	140 (61.9%)	1.026	0.862		
Distant metastasis^a^	96 (38.1%)	1.000			
Postsurgical adjuvant treatment					
Yes	175 (74.2%)	0.906	0.309		
No^a^	61 (25.8%)	1.000			
Postsurgical MTB_WB_ on PET/CT					
SUV_WBmax_	236 (100%)	1.066	< 0.001	1.052	0.002
MTV_WB_ (cm^3^)	236 (100%)	1.421	< 0.001	1.443	< 0.001
TLG_WB_ (cm^3^)	236 (100%)	1.301	0.001	1.332	0.005

Note. *OS* overall survival, *PET* positron emission tomography, *HR* hazard ratio, *CI* confidence interval, *PAC* papillary adenocarcinoma, *TAC* tubular adenocarcinoma, *PDAC* poorly differentiated adenocarcinoma, *SRC* signet-ring cell carcinoma, *MRC* mucinous adenocarcinoma, *SUV* standardized uptake value, *WB* whole body, *Max* maximum, *MTV* metabolic tumor volume, *TLG* total lesion glycolysis. ^a^, Reference group. Data in parentheses are percentages of corresponding variables

## Discussion

One problem of the various published studies about the utility of ^18^F-FDG PET/CT in patients with gastric cancer is that many attentions had been payed to preoperative PET/CT examination, with the postsurgical one being rarely studied [4, 11, 14, 15]. Clinical practice tells that there are a substantial portion of patients only underwent postsurgical PET/CT examination for tumor restaging or recurrence surveillance, and some of them even experienced repeated PET/CT scanning without anyone preformed preoperatively. In current study, only 649 (41.8%) of the 1554 patients with gastric cancer performed preoperative PET/CT, whereas more than a half patients underwent postoperative PET/CT. The potential value of postsurgical PET/CT to patients with gastric cancer require to be determined. In this study, we found that positive ^18^F-FDG uptake on first-time postsurgical PET/CT was an independent and significant prognostic factor for predicting overall survival of patient with gastric cancer after curative surgical resection. Similar results had been found in patients with lung cancer, previously [16]. These results suggested that postsurgical ^18^F-FDG PET/CT played an important role in predicting prognosis of gastric-cancer patients.

In addition, we demonstrated that higher MTB_WB_ indicated by increased SUV_WBmax_, MTV_WB_ and TLG_WB_ on postsurgical ^18^F-FDG PET/CT significantly correlated with worse OS in gastric-cancer patients. Similar associations had been reported between MTB_WB_ on pre-surgical ^18^F-FDG PET/CT and OS of patients with gastric cancer in previous study [14]. Based on early prognosis assessment using quantitative MTB_WB_ parameters on postsurgical PET/CT, it might be possible to select patients who require more aggressive treatment to improve their outcomes. In contrast, MTV_WB_ (HR = 1.443) and TLG_WB_ (HR = 1.322) presented better prognostic performance than SUV_WBmax_ (HR = 1.052). This may because SUV_WBmax_ does not contain volumetric information and thus fails to reflect whole-body burden of tumor, given that metastasis or recurrence of gastric cancer after operation is not always restricted to a single lesion. The high metabolic activity in one primary or metastatic site (SUV_WBmax_) may be not always associated with a large tumor burden.

Although the most aggressive focus within a tumor may be the most important in explaining the biological behavior of the entire tumor, total tumor volume and its metabolic activity are also interest and important when characterizing a tumor [19]. Therefore, volume-based parameters, such as MTV and TLG, have presented useful values in predicting prognosis and evaluating treatment response of various malignancies [11, 14–16, 20]. Previous studies have demonstrated that MTV and TLG had excellent sensitivity and specificity in predicting treatment response and survival outcomes [21, 22]. One problem of these volumetric parameters is that they cannot escape from influences of volume effects from adjacent lesions of high radioactivity, for example, the metastatic lymphonodus. With regard to this, the MTB_WB_ parameters, in terms of MTV_WB_ and TLG_WB_, may be more consistent and reproducible, as it is not necessary to separate primary tumors from adjacent lesions of high radioactivity but just need to delineate them together.

Previous studies have shown that preoperative ^18^F-FDG PET/CT has a low detection rate for primary gastric cancer [23, 24]. Normal physiological gastric activity, underlying inflammation, and wide range of metabolic activity of gastric cancer could be hurdles for accurate quantification. In contrast, ^18^F-FDG PET/CT performed after curative-intent operation is less influenced by these factors as the stomach has been totally or sub-totally resected. Thus, postoperative PET/CT may have better performance than its preoperative counterpart in predicting prognosis of gastric cancer patients. However, further studies are required to confirm this hypothesis. Limitation of postoperative FDG PET in gastric cancer imaging also exist. For example, the signet ring adenocarcinoma/mucinous adenocarcinoma tend to be FDG negative, but some patients with negative FDG uptake of the primary tumor could still have recurrent malignancy. This means a high percentage of recurrence might have been missed by FDG PET, especially in patient with primary tumor of this pathological type.

In this study, postsurgical treatment was identified negatively correlated with patients’ overall survival (HR = 1.552 and *P* = 0.001 for the whole subject cohort; Table 3), which seemed contradictive to the clinical reality. This might because patients who underwent postsurgical treatment had a high rate of positive PET finding, indicating a high tumor burden in these patients. When analyzed within the PET-positive patient group, postsurgical treatment turned to be a potential protective factor to patient overall survival (HR = 0.906), although not statistically significant (*P* = 0.306, Table 4). Further studies are required to determine the value of postsurgical treatment to patients with gastric cancer.

There are several limitations in this study. Firstly, validation of the threshold for volume-based PET/CT parameters was not performed. We just selected the widely used threshold for tumor-volume measurement. As volume-based parameters can be affected by the delineation method, multiple methods with different thresholds should have been compared. However, the intra- and inter-observer agreements of MTB_WB_ measurements were good, which guaranteed the reliability and reproducibility of the method in this study. Secondly, the patients included in this study had diverse time intervals between operation and PET/CT scanning (2–18 months), which might have introduced bias to the results of the study. However, we believe this bias was weak as time intervals from operation to PET/CT scanning were not statistically different between PET positive and negative patients. Finally, because this study was a retrospective single-center study, selection bias was inevitable. Thus, further multi-center studies are needed to confirm the results of our study.

## Conclusions

Postoperative ^18^F-FDG PET/CT of patients with gastric cancer had significant prognostic value in predicting patient overall survival. Patients with positive ^18^F-FDG uptake had significantly worse overall survival than patients with negative ^18^F-FDG uptake. Furthermore, the MTB_WB_ parameters on postoperative PET/CT, namely SUV_WBmax_, MTV_WB_ and TLG_WB_, independently predict overall survival of patients with gastric cancer after curative-intent operation.

## Abbreviations

HR: Hazard ratio
ICC: Intra-class correlation coefficient
MAC: Mucinous adenocarcinoma
MTB: Metabolic tumor burden
MTV_WB_: whole body metabolic tumor volume
OS: Overall survival
PAC: Papillary adenocarcinoma
PDAC: Poorly differentiated adenocarcinoma
PET/CT: Positron emission tomography/computed tomography
SRC: Signet-ring cell carcinoma
SUV_WBmax_: whole body maximum standard uptake value
TAC: well differentiated and moderately differentiated tubular adenocarcinoma
TLG_WB_: whole body total lesion glycolysis
VOI: Volume of interest
WB: Whole body
WB: Whole body^18^F-FDG 2-[18F] Fluoro-2-deoxy-D-glucose
